# Amino acid substitutions in the non-structural proteins 4A or 4B modulate the induction of autophagy in West Nile virus infected cells independently of the activation of the unfolded protein response

**DOI:** 10.3389/fmicb.2014.00797

**Published:** 2015-01-15

**Authors:** Ana-Belén Blázquez, Miguel A. Martín-Acebes, Juan-Carlos Saiz

**Affiliations:** ^1^Department of Biotechnology, Instituto Nacional de Investigación y Tecnología Agraria y AlimentariaMadrid, Spain; ^2^Department of Virology and Microbiology, Centro de Biología Molecular “Severo Ochoa” (CSIC-UAM), MadridSpain

**Keywords:** autophagy, LC3, West Nile virus (WNV), replication, host cells, unfolded protein response

## Abstract

West Nile virus (WNV) is a neurotropic mosquito-borne flavivirus responsible for outbreaks of meningitis and encephalitis. Whereas the activation of autophagy in cells infected with other flaviviruses is well known, the interaction of WNV with the autophagic pathway still remains unclear and there are reports describing opposite findings obtained even analyzing the same viral strain. To clarify this controversy, we first analyzed the induction of autophagic features in cells infected with a panel of WNV strains. WNV was determined to induce autophagy in a strain dependent manner. We observed that all WNV strains or isolates analyzed, except for the WNV NY99 used, upregulated the autophagic pathway in infected cells. Interestingly, a variant derived from this WNV NY99 isolated from a persistently infected mouse increased LC3 modification and aggregation. Genome sequencing of this variant revealed only two non-synonymous nucleotide substitutions when compared to parental NY99 strain. These nucleotide substitutions introduced one amino acid replacement in NS4A and other in NS4B. Using genetically engineered viruses we showed that introduction of only one of these replacements was sufficient to upregulate the autophagic pathway. Thus, in this work we have shown that naturally occurring point mutations in the viral non-structural proteins NS4A and NS4B confer WNV with the ability to induce the hallmarks of autophagy such as LC3 modification and aggregation. Even more, the differences on the induction of an autophagic response observed among WNV variants in infected cells did not correlate with alterations on the activation of the unfolded protein response (UPR), suggesting an uncoupling of UPR and autophagy during flavivirus infection. The findings here reported could help to improve the knowledge of the cellular processes involved on flavivirus–host cell interactions and contribute to the design of effective strategies to combat these pathogens.

## INTRODUCTION

West Nile virus (WNV) is a neurotropic mosquito-borne pathogen classified in the *Flaviviridae* family, genus *Flavivirus.* The viral genome is constituted by a single molecule of RNA of positive polarity about 11 kb in length that encodes three structural proteins and seven non-structural (NS) proteins (Martin-Acebes and Saiz, 2012). WNV is maintained in nature in an enzootic transmission cycle between avian hosts and ornithophilic mosquito vectors, but it can infect multiple vertebrate species, including humans and horses (Martin-Acebes and Saiz, 2012). Although infections in humans are mainly asymptomatic, WNV can also induce a wide range of clinical symptoms that varies from a mild flu-like febrile illness termed WN fever to a neuroinvasive disease characterized by meningitis, encephalitis, or acute flaccid paralysis (Hayes and Gubler, 2006). During the last years, research on WNV has been intensified but there is still no specific therapy or vaccine licensed for human use.

The replication of WNV relies on modified endoplasmic reticulum (ER) derived membrane structures (Gillespie et al., 2010; Martin-Acebes et al., 2011). Infection with WNV results in the induction of ER stress, which triggers a coordinated change in gene expression collectively known as unfolded protein response (UPR; Medigeshi et al., 2007; Ambrose and Mackenzie, 2011, 2013). As the UPR, autophagy (a cellular process by which cytoplasmic components are sequestered into double-membrane vesicles and degraded to maintain cellular homeostasis) also constitutes an evolutionarily ancient process for survival during different forms of cellular stress, including infection with viruses (Mizushima et al., 2008; Orvedahl and Levine, 2008). In this way, both the UPR and autophagy are two processes, sometimes interconnected, activated to cope with cellular stress (Suh et al., 2012). The induction of UPR and autophagy has been documented for multiple members of the *Flavivirus* genus, including Dengue virus (DENV), Japanese encephalitis virus (JEV), Usutu virus (USUV), or Modoc virus (reviewed in Blazquez et al., 2014; Green et al., 2014). However, to our knowledge, no direct relationship between activation of the UPR and autophagy has been assessed to date for WNV, or any other member of the *Flavivirus* genus. Even more, whereas the activation of the UPR following infection with WNV has been well documented (Medigeshi et al., 2007; Ambrose and Mackenzie, 2011, 2013), the induction of an autophagic response in WNV-infected cells still remains contentious, with evidences supporting both the upregulation (Beatman et al., 2012; Kobayashi et al., 2014) or not (Vandergaast and Fredericksen, 2012) of this pathway. Both autophagy and UPR constitute druggable metabolic pathways under evaluation for multiple therapeutic interventions (Suh et al., 2012; Cao and Kaufman, 2013). Deciphering interactions with these cellular mechanisms could help to the development of novel antiviral strategies.

In this study, we have analyzed the induction or not of autophagy and the UPR in cells infected with different WNV variants. Our results showed that point mutations in the viral non-structural proteins NS4A and NS4B, which are involved in WNV-induced membrane rearrangements (Roosendaal et al., 2006; Kaufusi et al., 2014), could modulate the ability to induce the characteristic features of an autophagic response in infected cells. These findings shed new light in this topic, thus helping to explain the previous contradictory reports related to the ability of WNV to promote or not an autophagic response. On the other hand, our results also showed that alterations on the ability to induce an autophagic response did not correlate with alterations on the induction of the UPR, suggesting an uncoupling of the UPR and autophagy during flavivirus infection.

## MATERIAL AND METHODS

### ANTIBODIES

Mouse monoclonal antibody J2 against double-stranded RNA (dsRNA; English & Scientific Consulting), rabbit monoclonal anti-LC3B (Sigma or Cell Signaling) and mouse monoclonal anti-β-actin (Sigma), were used as primary antibodies. Secondary antibody against mouse IgGs coupled to Alexa Fluor-594 (Life Technologies) was used for immunofluorescence assays. Anti-rabbit (Dako) and anti-mouse (Sigma) secondary antibodies coupled to horseradish peroxidase were used in western blot assays.

### CELLS, VIRUSES, INFECTIOUS cDNA CLONE MANIPULATION AND INFECTIONS

All manipulations of infectious virus were carried out in Biosafety level 3 (BSL-3) containment facilities. The WNV and USUV used have been summarized in **Table 1**. Recombinant WNV were recovered from infectious cDNA clone pFLWNV (Shi et al., 2002), that contained the full length sequence of a North American isolate of WNV [termed Wild type (WT)], or from its derivatives containing the nucleotide substitutions G6667A (NS4A V67I), A7635G (NS4B I240 M) or both combined. Nucleotide substitutions were introduced into pFLWNV by site-directed mutagenesis using QuikChange II XL (Agilent) as described (Martin-Acebes et al., 2013) and oligonucleotide primers shown on **Table 2**. Viruses were recovered from infectious clones by *in vitro* transcription and transfection of viral RNA (Martin-Acebes et al., 2013). All virus stocks used in the experiments were produced by infection of Vero cells. Cells were washed with Eagle’s minimal essential medium (EMEM) before the addition of the inoculum. Viruses were used at a multiplicity of infection (MOI) of 5 PFU/cell in fluorescent microscopy experiments and of 0.5 PFU/cell in the rest of experiments. Virus titrations on semisolid agar medium were performed as previously described (Martin-Acebes and Saiz, 2011). Cells were routinely tested for mycoplasma with Mycoalert Mycoplasma Detection Kit (Lonza).

**Table 1:** Virus strains, isolates and infectious cDNA clone used in the study.

Virus	Strain/isolate	Relevant data
WNV	NY99	North American strain isolated in New York in 1999 (Lanciotti et al., 1999; Martin-Acebes and Saiz, 2011)
	B13	Persistent variant of NY99 isolated from a mice 56 days after vertical infection (Blazquez and Saiz, 2010)
	ArD27875	Isolated in Senegal in 1990 (Beasley et al., 2002)
	Egypt101	Isolated in Egypt in 1950 (Beasley et al., 2002)
	B956	Isolated in Uganda in 1937 (Smithburn et al., 1940)
	pFLWNV (WT)	Infectious cDNA clone containing a derivative of the North American strain 3356 isolated in New York in 2000 (Shi et al., 2002)
USUV	SAAR 1776	South African reference strain (Bakonyi et al., 2004)

**Table 2:** Oligonucleotide primers used for site-directed mutagenesis.

Mutant	Orientation	Primer sequence^a^
NS4A V67I	Forward	GCCTTATTGAGTGTGATGACCATGGGA**A**TAT TCTTCCTCCTCATGCAGCGGAAGGGC
	Reverse	GCCCTTCCGCTGCATGAGGAGGAAGAATA**T** TCCCATGGTCATCACACTCAATAAGGC
NS4B I240M	Forward	GGGGGTTGGTTGTCATGTCTATCCAT**G**ACA TGGACACTCATAAAGAACATGGAAAAACC
	Reverse	GGTTTTTCCATGTTCTTTATGAGTGTCCATGT **C**ATGGATAGACATGACAACCAACCCCC

*^a^Mutated positions relative to NY99 isolate (KC407666) are shown in bold.*

### DRUG TREATMENTS

Tunicamycin (Sigma), an inducer of UPR, was dissolved in DMSO and used at 10 μg/ml. Salubrinal (Santa Cruz), an inhibitor of UPR was dissolved in DMSO and used at 5 μg/ml. 3-methyladenine (3-MA; Sigma), an inhibitor of autophagy was used at 2.5 mM. Drugs were added to the medium after virus adsorption and the same volume of drug vehicle was added as control in non-treated cells. Protease inhibitors E-64d and pepstatin A (10 μg/ml each; Sigma) were added to cell culture medium 4 h before cells were harvested for western blot analysis (Klionsky et al., 2012). The viability of cells with or without treatment was tested with CellTiter-Glo Luminiscent Cell Viability Assay (Promega).

### ANALYSIS OF LC3 MODIFICATION AND AGGREGATION

Autophagosome formation was determined as described (Blazquez et al., 2013). Briefly, a plasmid encoding GFP-LC3 (Kabeya et al., 2000) was transfected using Fugene HD (Promega). Cells were infected 24 h post-transfection and fixed and processed for immunofluorescence (Martin-Acebes et al., 2011; Blazquez et al., 2013). Samples were observed using a Leica TCS SPE confocal laser-scanning microscope. Images were acquired using Leica Advanced Fluorescence Software. LC3 dots were counted at least for 30 different cells corresponding to each virus and treatment using ImageJ software (http://imagej.nih.gov/ij/). To this end, the fluorescence in the green channel was processed using a mean filter (2.0 pixels) and a threshold was applied to select LC3 dots in each cell. Then, the number of LC3 aggregates was automatically determined using Analyze particles tool. Images were processed using ImageJ and Adobe Photoshop CS2. Detection of LC3-I and II by western blot was performed as reported (Martin-Acebes et al., 2011; Blazquez et al., 2013). β-actin was also detected by western blot as control for protein loading.

### DETECTION OF Xbp-1 mRNA

RNA extraction and detection of spliced and unspliced X box binding protein 1 (Xbp-1) by RT-PCR was performed as previously described (Blazquez et al., 2013). Amplification of GAPDH mRNA was carried as a control for RNA extraction (Blazquez et al., 2013). PCR products were resolved by electrophoresis in a 2% agarose gel.

### STATISTICAL ANALYSES

Data are presented as mean ± SD. To test the significance of the differences, analysis of the variance (ANOVA) and Student’s *t*-test was performed with statistical package SPSS 15 (SPSS Inc.) applying Bonferroni’s correction for multiple comparisons. Statistically significant differences were considered at *P* < 0.05.

## RESULTS

### DIFFERENT AUTOPHAGIC FEATURES IN CELLS INFECTED WITH DIVERSE WNV VARIANTS

**FIGURE 1:**
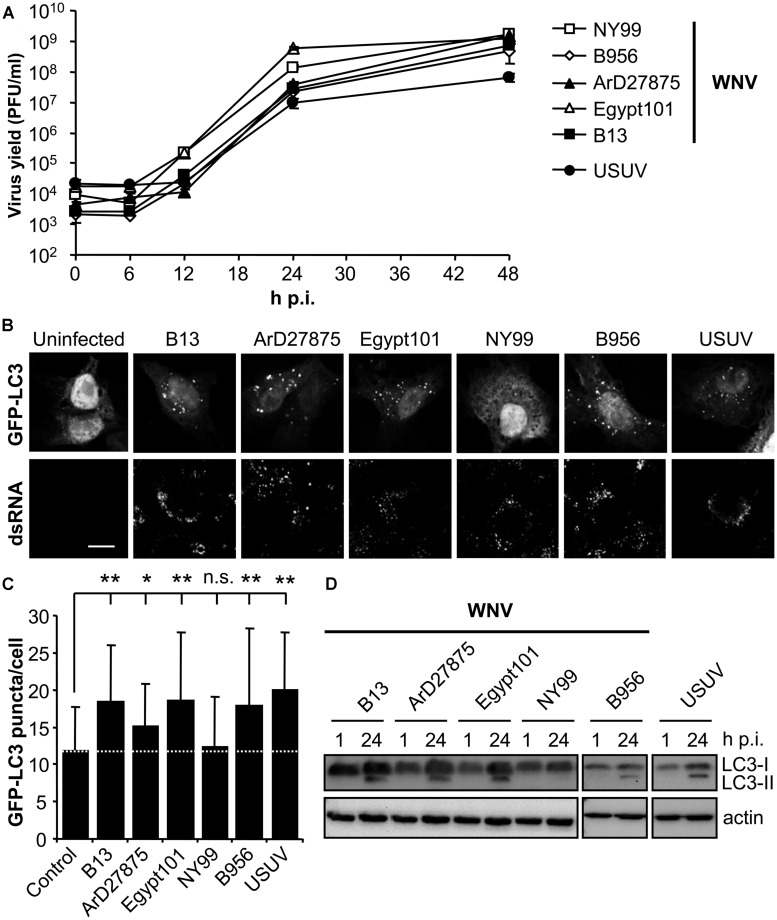
**Differences on the induction of LC3 modification and aggregation in cells infected with diverse variants of West Nile virus (WNV). (A)** Growth curves of WNVs in Vero cells. Cells were infected (MOI of 0.5 PFU/cell) with WNVs (B13, B956, ArD27875, Egypt101, and NY99) or USUV and supernatant virus yield was determined at different times p.i. by standard plaque assay on Vero cells. **(B)** Visualization of autophagosome formation by LC3 aggregation in cells infected with the viruses displayed in panel **(A)**. Vero cells were transfected with a plasmid encoding GFP-LC3 and 24 h post-transfection were infected with WNV or USUV (MOI of 5 PFU/cell). Cells were fixed and processed for immunofluorescence using a monoclonal antibody against dsRNA and secondary antibodies AF-594 labeled 24 h p.i. Scale bars: 10 μm. **(C)** Quantification of the number of LC3 aggregates per cell. The number of fluorescent aggregates on the cytoplasm of cells infected in **(B)** was determined. Dashed line indicates the mean number of GFP puncta aggregates found in uninfected cells. Statistically significant differences between infected and uninfected cells are denoted by one asterisk (*) for *P* < 0.05 or two asterisks (**) for *P* < 0.005. n.s. denotes not statistically significant differences. **(D)** Monitoring LC3 modification following infection by WNV or USUV. Vero cells infected with different WNVs or USUV (MOI of 0.5 PFU/cell) were lysed at 1 or 24 h p.i. and subjected to western blot analysis using an antibody against LC3 to detect non-lipidated LC3 (LC3-I) and LC3 conjugated to phosphatidylethanolamine (LC3-II). An anti-β-actin antibody was also used as control for protein loading.

To analyze the possible upregulation of the autophagic pathway in WNV-infected cells, a panel of WNVs that varied temporally and geographically was selected (**Table 1**). The related flavivirus USUV, whose ability to induce an autophagic response and to induce the UPR has been previously documented (Blazquez et al., 2013), was also included in the analyses as a positive control (**Table 1**). All these viruses shared similar growth kinetics in Vero cells, enabling further comparisons among them (**Figure 1A**). Microtubule-associated protein 1 light chain 3 (LC3) is post translationally modified by conjugation to phosphatidylethanolamine and targeted to autophagic membranes and, thus, this protein constitutes a widely used marker to analyze the upregulation of the autophagic pathway (Klionsky et al., 2012). The aggregation of LC3 in autophagic vacuoles can be observed by fluorescence microscopy as an increase in LC3 puncta in the cell cytoplasm (Kabeya et al., 2000; Klionsky et al., 2012). Hence, Vero cells were transfected with a plasmid encoding GFP-LC3 (Kabeya et al., 2000) for 24 h, then infected with the different viruses and fixed 24 h postinfection (p.i.). Cells were subjected to immunofluorescence analysis using an antibody directed against dsRNA, to confirm that transfected cells were infected (Gillespie et al., 2010; Martin-Acebes et al., 2011; **Figure 1B**). Uninfected cells exhibited GFP-LC3 fluorescence in both nucleus and cell cytoplasm, and low amounts of GFP-LC3 aggregates throughout the cytoplasm. As expected, no signal of dsRNA was noticed in uninfected cells. On the other hand, cells infected with USUV were positive for dsRNA and displayed a redistribution of GFP-LC3 fluorescence toward cytoplasmic puncta that likely corresponded to autophagosomes (Blazquez et al., 2013), thus confirming that the system worked properly. In the case of WNV variants, infection with B13, ArD27875, Egypt101, or B956 also resulted in the aggregation of GFP-LC3 fluorescence in cytoplasmic structures. However, cells infected with NY99 did not redistribute the GFP-LC3 signal. The amount of GFP-LC3 puncta per cell was determined by confocal microscopy and it was found that the increase in GFP-LC3 aggregates in cells infected with WNVs B13, ArD27875, Egypt101, and B956, or with USUV, was statistically significant compared to uninfected cells (**Figure 1C**). In the case of cells infected with NY99, no statistically significant differences were observed relative to uninfected cells, indicating a differential behavior of this viral isolate (**Figure 1C**). Next, the modification of LC3 following infection was analyzed by western blot. To this end, cells were infected with the different viruses and lysed at 1 or 24 h p.i. An increase in the amount of the lipidated form of LC3 (termed LC3-II) that displays a higher relative mobility than the non-lipidated form (termed LC3-I; Klionsky et al., 2012) was observed in cells infected with WNVs B13, ArD27875, Egypt101, and B956, or with USUV, but not in those cells infected with NY99. Overall, these results indicate a differential induction of autophagic response between NY99 and the other WNV variants analyzed.

### COMPARISON OF DIFFERENT AUTOPHAGIC FEATURES BETWEEN WNV NY99 AND B13 VARIANTS

**FIGURE 2:**
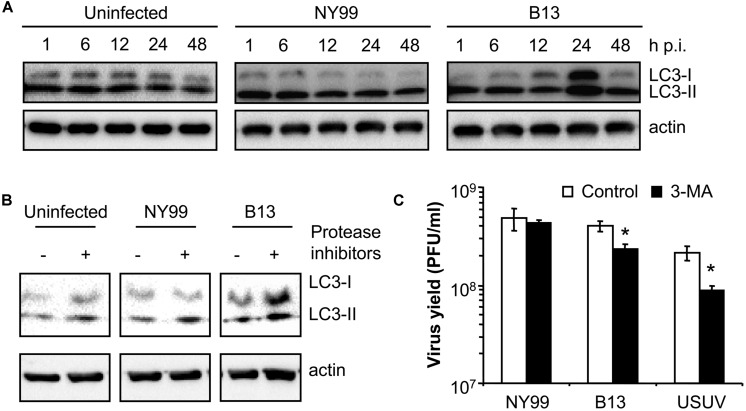
**Characterization of the differences between WNV NY99 and B13 variant. (A)** Time course analysis of LC3 modification following infection by WNV NY99 or B13. Vero cells infected with WNV NY99 or B13 (MOI of 0.5 PFU/cell) were lysed at 1, 6, 12, 24, or 48 h p.i. and subjected to western blot analysis using an antibody against LC3 to detect non-lipidated LC3 (LC3-I) and LC3 conjugated to phosphatidylethanolamine (LC3-II). An anti-β-actin antibody was also used as control for protein loading. Mock-infected cells were analyzed in parallel to show the levels of LC3-II in uninfected cells. **(B)** Analysis of LC3 modification following infection by WNV NY99 or B13 including protease inhibitors (E-64d and pepstatin A). Vero cells were infected with WNV NY99 or B13 (MOI of 5 PFU/cell) and harvested at 24 h p.i. Protease inhibitors E-64d and pepstatin A (10 μg/ml each) were added to culture medium 4 h before harvesting the cells. Mock-infected cells were analyzed in parallel to show the effect of inhibitors in uninfected cells. Western blot analysis was performed as described for panel A. **(C)** Effect of pharmacological inhibition on the infection of WNV NY99 and B13. Cells infected with the different viruses (MOI of 0.5 PFU/cell) were treated with 2.5 mM 3-MA and the virus yield were determined by plaque assay at 24 h p.i. USUV was included as a positive control. Statistically significant differences between each condition and control cells are denoted by one asterisk (*) for *P* < 0.05.

Because the autophagic induction is a dynamic process, and to rule out that the differences above commented were product of different kinetics of LC3 modification, a time course analysis of infection was performed (**Figure 2A**) with NY99 strain and its derivative B13 variant (**Table 1**). An increase in the amount of LC3-II was patent at 24 h in cells infected with B13 that was not noticed in mock-infected cells or in cultures infected with NY99 at 1, 6, 12, 24, or 48 h p.i., thus confirming that differences on LC3-II accumulation were not related to different viral infection kinetics.

To exclude that the increase in LC3-II content in B13 infected cells was the result of inhibition of the degradation of autophagosomes instead of an upregulation of autophagy, cells were treated with protease inhibitors E-64d and pepstatin (Klionsky et al., 2012) and the levels of LC3-II were analyzed at 24 h p.i. When compared to those cells not treated with the inhibitors, an increase in LC3-II in uninfected cells treated with the inhibitors was observed (**Figure 2B**). This is consistent with an accumulation of LC3-II as a result of the blockage of the normal autophagic flux and confirmed that the inhibitors worked under the experimental settings. In the case of NY99-infected cells, also a similar increase in LC3-II was detected upon treatment with protease inhibitors, as previously reported (Vandergaast and Fredericksen, 2012). However, in the case of B13 infected cells, the increase in LC3-II was more marked in the cells treated with the inhibitors. Hence, these results supported the differential behavior between NY99 and B13.

Next, the effect of autophagy inhibitor 3-MA (Klionsky et al., 2012) was also analyzed on the infection of NY99, B13, and USUV (**Figure 2C**) using a drug concentration (2.5 mM 3-MA) that had been reported not to induce major toxic effects on Vero cells but enough to reduce the infection of USUV (Blazquez et al., 2013). Treatment with 3-MA induced a significant reduction the virus yield of B13 and USUV, but not that of NY99. Overall, these results further evidence the differences on the interaction with the autophagic pathway between NY99 and B13.

### MUTATIONS IN WNV NS4A AND NS4B PROTEINS MODULATE THE INDUCTION OF AUTOPHAGY

**FIGURE 3:**
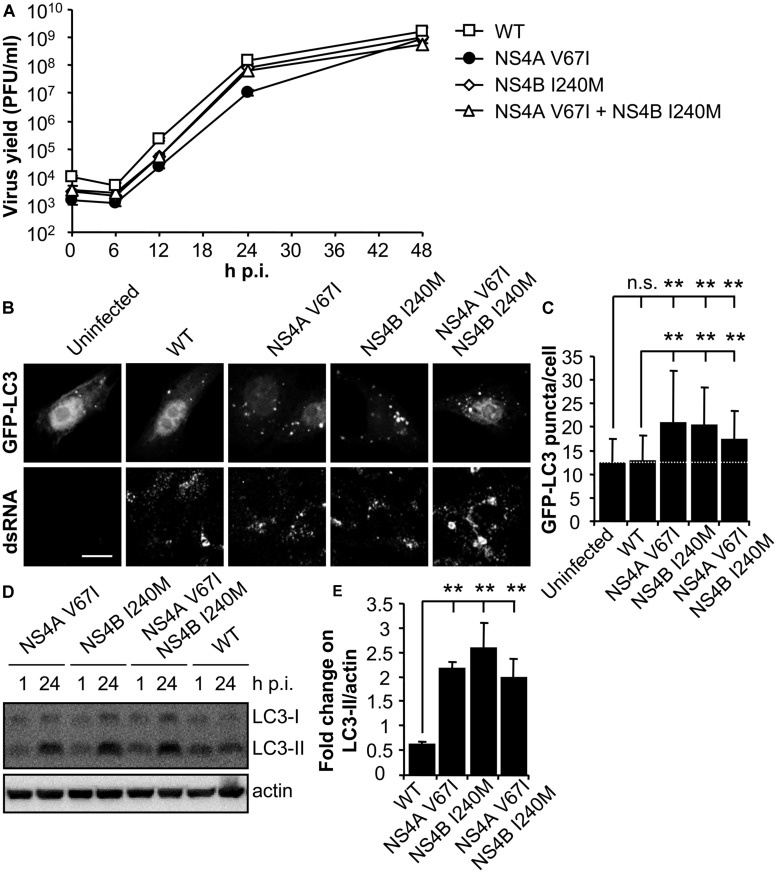
**Mutations on WNV NS4A or NS4B can modulate the induction of LC3 modification and aggregation in cells infected with WNV. (A)** Growth curves of recombinant WNV recovered from infectious cDNA clone pFLWNV (WT) or its derivatives carrying amino acid substitutions NS4A V67I, NS4B I240M or NS4A V67I + NS4B I240M. Vero cells were infected (MOI of 0.5 PFU/cell) with the viruses and the supernatant virus yield was determined at different times p.i. **(B)** Visualization of autophagosome formation by LC3 aggregation in cells infected with the viruses displayed in panel **(A)**. Vero cells were transfected with a plasmid encoding GFP-LC3 and 24 h post-transfection were infected (MOI of 5 PFU/cell), fixed and processed for immunofluorescence analysis as described in the legend for **Figure 1**. Scale bars: 10 μm. **(C)** The number of fluorescent aggregates on the cytoplasm of cells infected in **(B)** was determined. Dashed line indicates the mean number of GFP puncta aggregates found in uninfected cells. **(D)** Analysis of LC3 modification following infection with recombinant WNVs. Vero cells were infected (MOI of 0.5 PFU/cell) and lysed at 1 or 24 h p.i. Western blot analysis was performed as described in the legend of **Figure 1**. **(E)** Quantification of the fold change on LC3-II/actin from 1 to 24 h p.i. Results are product of three independent western blots similar to that displayed in panel **(D)**. Statistically significant differences between infected and uninfected cells, or cells infected with WT and mutant viruses, are denoted by two asterisks (**) for *P* < 0.005. n.s. denotes not statistically significant differences.

RNA from the parental NY99 and its derivative B13 variant was extracted from infected culture supernatants and their complete genomic sequence determined by nucleotide sequencing and compared. The genome of B13 (GenBank acc.: KC407667) carried only two nucleotide substitutions (G6667A and A7635G) when compared to the parental NY99 (GenBank acc.: KC407666). These nucleotide substitutions were responsible for the introduction of the amino acid replacements V67I in NS4A and I240M in NS4B, respectively. The nucleotide substitutions were introduced alone, or combined, into the infectious cDNA clone of WNV pFLWNV (**Table 1**), which contains the sequence of a highly related WNV isolate showing 99.82% sequence identity with the parental NY99 isolate used in this study. Virus recovered from pFLWNV infectious cDNA clone has been previously reported not to upregulate the autophagic pathway in infected cells (Vandergaast and Fredericksen, 2012), as observed for NY99 isolate used in this study. Therefore, these characteristics make of this clone a suitable genetic backbone to introduce the mutations found here to be candidates to act as autophagy modulators. Recombinant WNVs carrying amino acid substitutions recovered from infectious clones (Martin-Acebes et al., 2013) displayed similar growth kinetics in Vero cells (**Figure 3A**). WT virus recovered from pFLWNV did not increase the amount of GFP-LC3 puncta in infected cells (**Figure 3B**), as shown for NY99 (**Figure 1**). However, cells infected with mutant viruses carrying single amino acid replacement NS4A V67I or NS4B I240M exhibited an increase in GFP-LC3 aggregation. Similar features were also observed for the double mutant NS4A V67I +NS4B I240M (**Figure 3B**). In fact, when the number of GFP-LC3 puncta was determined, a statistically significant increase of LC3 aggregates was observed in cells infected with mutant viruses carrying NS4A V67I, NS4B I240M, or both substitutions combined relative to uninfected cells or cells infected with WT virus recovered from parental infectious cDNA clone (**Figure 3C**). In addition, no significant differences between cells infected with WT and uninfected cells were noticed, supporting the results observed for NY99 strain (**Figure 1C**). Even more, mutant viruses carrying NS4A V67I, NS4B I240M, or both substitutions combined, significantly increased the amount of LC3-II, in comparison to cells infected with WT virus (**Figures 3D,E**). Taken together, these results indicate that single amino acid substitutions in NS proteins are sufficient to modulate the interaction of WNV with the autophagic pathway.

### UPR INDUCTION AND AUTOPHAGY DO NOT EXHIBIT A CAUSE-EFFECT RELATIONSHIP IN WNV INFECTED CELLS

**FIGURE 4:**
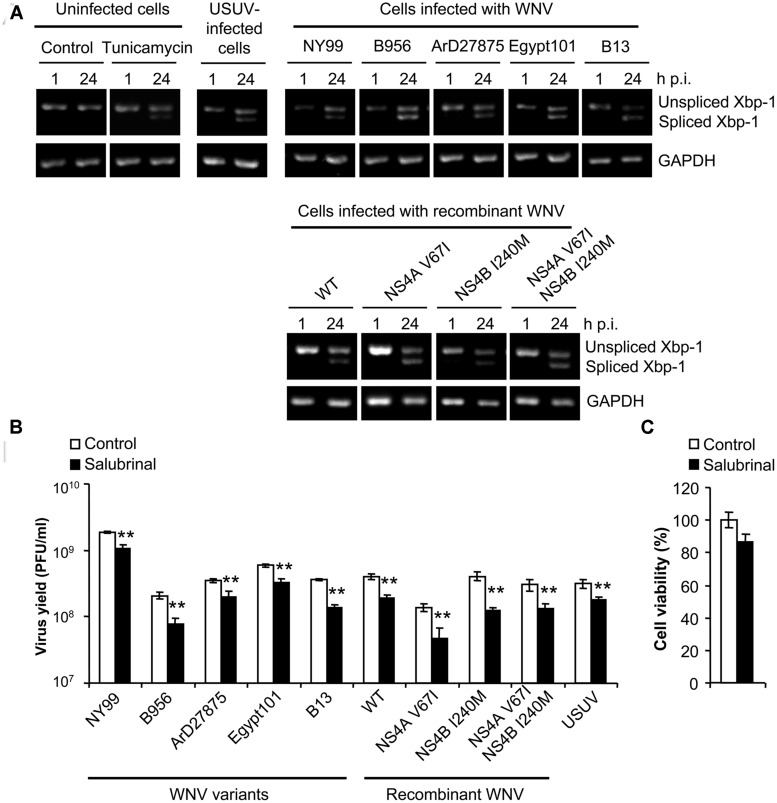
**Infection with WNV variants that differ in induction of autophagy results in similar UPR features. (A)** Activation of the unfolded protein response (UPR) following infection with WNV or USUV assayed by detection of spliced Xbp-1 mRNA. RNA was extracted from Vero cells infected with WNV B956, ArD27875, Egypt101, NY99, B13, recombinant WNV recovered from infectious cDNA clone pFLWNV (WT) or its derivatives carrying amino acid substitutions NS4A V67I, NS4B I240M or NS4A V67I + NS4B I240M, and the presence of unspliced or spliced Xbp-1 mRNA was determined by RT-PCR. Cells treated with tunicamycin, or infected with USUV are included as a positive control of the activation of UPR. GAPDH mRNA was also amplified by RT-PCR as a control. **(B)** Effects of pharmacological modulation of UPR with salubrinal on WNV infection. Cells infected (MOI of 0.5 PFU/cell) with the different viruses described in **(A)** were treated with 5 μM salubrinal or the same amount of drug vehicle (DMSO, control) and the virus yield was determined by plaque assay at 24 h p.i. **(C)** Analysis of cellular viability upon treatment with salubrinal. Vero cells were treated with 5 μM salubrinal or drug vehicle (DMSO, control) for 24 h and the viability was estimated by determination of cellular ATP levels by a luminescence assay. Statistically significant differences between each condition and control cells are denoted by two asterisks (**) for *P* < 0.005.

The splicing of Xbp-1 mRNA allows the expression of the full length transcription factor Xbp-1. The expression of this factor upregulates transcription of multiple genes aimed to cope with ER stress and has been reported as a common feature of UPR in WNV-infected cells (Medigeshi et al., 2007; Ambrose and Mackenzie, 2011). As shown on **Figure 4A**, and consistent with previous reports (Blazquez et al., 2013), cells treated with tunicamycin [a pharmacological inducer of UPR (Kohno et al., 1993)] or infected with USUV [a viral inducer of UPR (Blazquez et al., 2013)], displayed an increase in the amount of spliced Xbp-1 not observed in control cells, as did cells infected with all WNVs included in the study. Treatment with salubrinal, that protects cells from ER stress (Boyce et al., 2005), significantly reduced (40–50%) the infection by all viruses tested (**Figure 4B**) without exerting a toxic effect (**Figure 4C**). Since no major differences were noticed on the induction of Xbp-1 splicing, or the response to salubrinal, among viruses that upregulated the autophagic pathway (B956, ArD27875, Egypt101, B13, NS4A V67I, NS4B I240M or NS4A V67I, +NS4B I240M) when compared to those that did not (NY99 or pFLWNV WT), these results suggest that the induction of UPR does not constitute the major mechanism for upregulation of the autophagic pathway in cells infected with WNV.

## DISCUSSION

Autophagy and UPR, which are activated to cope with cellular stress, can be activated during viral infections. Since both pathways are involved in the host immune defense, these topics are becoming critical areas in antiviral research (Jheng et al., 2014). In fact, the modulation of autophagy or UPR could constitute a feasible antiviral approach against flaviviruses (Shoji-Kawata et al., 2013; Fraser et al., 2014). However, the connections between these two cellular pathways still remain obscure for most viruses (Jheng et al., 2014), including the flaviviruses (Blazquez et al., 2014).

Whereas induction of the UPR in WNV-infected cells has been well documented (Medigeshi et al., 2007; Ambrose and Mackenzie, 2011, 2013), controversial reports on the induction or not of autophagic features in WNV-infected cells have been published, including opposite results observed with different isolates from the same viral strain (NY99; Beatman et al., 2012; Vandergaast and Fredericksen, 2012; Kobayashi et al., 2014). Two different reports described an upregulation of autophagy in cells infected with NY99 isolates (Beatman et al., 2012; Kobayashi et al., 2014). However, another study by (Vandergaast and Fredericksen, 2012) using WNV NY99 recovered from pFLWNV (the same infectious clone used in this study) showed that WNV replication did not upregulate the autophagy pathway. Even more, whereas (Beatman et al., 2012) pointed that WNV growth was independent of autophagy, (Kobayashi et al., 2014) have recently proposed that autophagy negatively regulates WNV growth. Considering our results obtained with NY99 and its B13 variant, which differed only in two nucleotides, small differences on the nucleotide sequence of the viruses used in the different studies could be responsible of the opposite observations reported. In fact, differences on the ability to upregulate the autophagic pathway have been also reported for other flaviviruses (Li et al., 2012). The results obtained here with B13 variant and its derivatives recovered form infectious clones showed that minimal genetic changes between WNV strains were sufficient to promote the modification and aggregation of LC3. In this way, a single amino acid substitution in NS4A (V67I) or NS4B (I240M) protein was enough to modulate the interaction of WNV NY99 with the autophagic pathway. Hence, results here presented could explain the previous differences observed by other authors when examining close related viral isolates, as the product of minimal changes between their genomes. These results also contributed to map the genetic basis for the induction of autophagy in WNV proteins NS4A and NS4B. These are two viral proteins with multiple transmembrane elements that have been related to flavivirus-induced membrane rearrangements (Roosendaal et al., 2006; Miller et al., 2007; Kaufusi et al., 2014) and UPR activation (Ambrose and Mackenzie, 2011). These findings are compatible with results obtained with DENV, whose ability to upregulate the autophagic pathway has been associated with the expression of NS4A protein (McLean et al., 2012).

The activation of UPR by members of the *Flaviviridae* family has been related with the induction of an autophagic response (Sir et al., 2008; Ke and Chen, 2011; Wang et al., 2014), although other studies do not support this cause-effect relationship (Mohl et al., 2012). The UPR has three branches initiated by the stress sensors protein kinase RNA-like ER kinase (PERK), inositol-requiring protein 1 (IRE1) and activating transcription factor 6 (ATF6; Hetz, 2012; Blazquez et al., 2014). The activation of these three arms has been reported for WNV (Medigeshi et al., 2007; Ambrose and Mackenzie, 2011, 2013). Regarding the PERK arm, the role of the phosphorylation of eukaryotic initiation factor-2α (EIF2α), a key regulator of this pathway, was evaluated in this study by using salubrinal, which selectively inhibits EIF2α dephosphorylation and protects cells from ER stress (Boyce et al., 2005). No major differences on the inhibitory effect of salubrinal were observed among the different WNV analyzed, thus suggesting that differences on the PERK/EIF2α arm of UPR were not behind the differential upregulation of the autophagic pathway observed. In addition, the splicing of Xbp-1 was also analyzed and no major differences were found between the viral variants tested. The splicing of Xbp-1 correlates with activation of the IRE1 arm of UPR (Hetz, 2012). In addition, previous studies have revealed that during WNV infection there is also a crosstalk between ATF6 and IRE1 pathway that converge in Xbp-1 (Ambrose and Mackenzie, 2011). This crosstalk is also consistent with reports observed in other model systems (Yoshida et al., 2001). In this way, Xbp-1 acts as an indicator of UPR activation, which probably indicates both IRE1 and ATF6 activation during WNV infection. Hence, although the specific contribution of each branch of the UPR to the autophagic pathway in WNV remains to be analyzed in detail, the results here presented suggest that the differences on the induction of autophagy in WNV-infected cells here described do not seem to be induced by a different activation of UPR.

In this study we have contributed to map the genetic determinants of autophagy regulation in WNV-infected cells, thus helping to clarify the controversy over the induction or not of an autophagic response following infection with this flavivirus. Along this line, the findings here reported could help to improve the knowledge of the cellular processes involved on WNV-host cell interactions contributing to the design of effective strategies to combat this pathogen.

## Conflict of Interest Statement

The authors declare that the research was conducted in the absence of any commercial or financial relationships that could be construed as a potential conflict of interest.
